# Intercellular adhesion molecule 2 regulates diapedesis hotspots by allowing neutrophil crawling against the direction of flow

**DOI:** 10.1530/VB-23-0005

**Published:** 2023-08-31

**Authors:** Max L B Grönloh, Merel E Tebbens, Marianthi Kotsi, Janine J G Arts, Jaap D van Buul

**Affiliations:** 1 Department of Medical Biochemistry, Vascular Biology Lab, Amsterdam UMC, University of Amsterdam, Amsterdam, the Netherlands; 2 Leeuwenhoek Centre for Advanced Microscopy, Section Molecular Cytology at Swammerdam Institute for Life Sciences, the University of Amsterdam, Amsterdam, the Netherlands; 3 Department of Molecular Hematology, Sanquin Research, and Landsteiner Laboratory, Molecular Cell Biology Lab, Amsterdam, the Netherlands

**Keywords:** transendothelial migration, neutrophils, endothelium, ICAM-2, blood flow

## Abstract

Intercellular adhesion molecules (ICAMs) are cell surface proteins that play a crucial role in the body’s immune response and inflammatory processes. ICAM1 and ICAM2 are two ICAM family members expressed on the surface of various cell types, including endothelial cells. They mediate the interaction between immune cells and endothelial cells, which are critical for the trafficking of leukocytes across the blood vessel wall during inflammation. Although ICAM1 plays a prominent role in the leukocyte extravasation cascade, it is less clear if ICAM2 strengthens ICAM1 function or has a separate function in the cascade. With CRISPR–)Cas9 technology, endothelial cells were depleted for ICAM1,ICAM2, or both, and we found that neutrophils favored ICAM1 over ICAM2 to adhere to. However, the absence of only ICAM2 resulted in neutrophils that were unable to find the transmigration hotspot, i.e. the preferred exit site. Moreover, we found that ICAM2 deficiency prevented neutrophils to migrate against the flow. Due to this deficiency, we concluded that ICAM2 helps neutrophils find the preferred exit sites and thereby contributes to efficient leukocyte extravasation.

## Introduction

During inflammation, leukocytes migrate through the endothelial monolayer in a process called transendothelial migration (TEM). TEM is a multistep process, consisting of several well-defined steps that take place in a sequential manner: rolling, firm adhesion, crawling, and finally diapedesis (1, 2, 3, 4, 5).

It is well recognized that a wide range of factors such as junction phenotype (6), substrate stiffness (7), and chemokine gradients (8) can impact the capability of leukocytes to exit the vasculature at certain spots (9). Recently, research has demonstrated that leukocyte extravasation does not take place at random sites, on the endothelium, but at the so-called hotspot areas (10, 11, 12). Physiologically, these TEM hotspot regions exist to maintain vascular barrier integrity, by allowing only a small part of the endothelial monolayer to be penetrated by extravasating leukocytes (11). Additionally, heterogeneous expression of intercellular adhesion molecule (ICAM) 1, an inflammation-induced transmembrane protein (13), was revealed to regulate hotspots in inflamed endothelial monolayers, as endothelial cells (ECs) expressing high levels of ICAM1 correlated to neutrophil adhesion hotspots (11). These data were in line with intravital FRET-imaging performed in mice, which showed important roles of well-known ICAM1 binding partners lymphocyte function-associated antigen 1 (LFA-1; CD11a/CD18) and macrophage-1 antigen (MAC-1; CD11b/CD18) during extravasation at hotspot sites (10). However, ICAM1 is not the only binding partner of LFA-1 and MAC-1. ICAM2, which is constitutively expressed in noninflamed ECs, also binds both these integrins (14, 15). Previous research from our group focused on the role of ICAM1 in the regulation of hotspots, but we also showed that ICAM-2^high^ cells are used by adhering neutrophils to cross the endothelium. This became apparent in endothelial cells that lacked any ICAM1 expression (11). These data suggest that there might be a yet unrevealed role for ICAM2 in the regulation of TEM hotspots.

ICAM1 and ICAM2 bind integrins via their extracellular immunoglobulin (Ig)-like domains, of which they have five and two, respectively (16, 17, 18). The bindings modulated by these domains allow ICAM1 and ICAM2 to be involved mainly in the firm adhesion and crawling phases of TEM (19, 20). So far, LFA-1 binding has mainly been associated with the firm adhesion step, and MAC-1 has been implicated in the crawling step of TEM (21). However, the exact roles of these proteins are most likely different between leukocyte subsets, as well as between types of endothelial beds (22, 23, 24). Intracellularly, ICAM1 has been shown to interact with actin-adaptor proteins α-actinin (25), filamin B (26), and cortactin (27), leading to actin modulation downstream of ICAM1 clustering. ICAM2 has also been demonstrated to regulate actin assembly, via proteins such as α-actinin (28) or the Ezrin/Radixin/Moesin (ERM) protein family (29). The similarity in the type of proteins both ICAMs interact with does suggest a partial overlap in their function, but this has not been extensively studied yet. However, previous research did demonstrate that a double knockout of both ICAMs was able to decrease neutrophil adhesion on human umbilical vein endothelial cells (HUVECs) (11), and neutrophil crawling on mouse inflamed endothelium and blood–brain barrier (BBB) ECs (6, 19).

In this study, we made use of ICAM1, ICAM2, and double knockout (KO) endothelial cells to show that that endothelial cells depleted for ICAM2 have reduced number of TEM hotspots and show a more random TEM pattern. Moreover, using ICAM2 truncated proteins, we showed that in the presence of ICAM1, ICAM2 is not required for adhesion or diapedesis efficacy of neutrophils. However, we show that both the first Ig-like domain and the intracellular domain of ICAM2 are required for neutrophils to crawl against the direction of flow. In conclusion, we show that ICAM2 has an additional role in the inflammatory TEM cascade by mediating strong adhesion of the leukocyte to the endothelium, allowing the leukocyte to crawl against the flow to find the TEM hotspot.

## Methods

### Plasmids

pLV-mScarlet-CaaX was described previously by our group (12). pLV-ICAM-2-mKate was ordered at VectorBuilder (Vector ID: VB200624-1164vtm). To generate ICAM2 truncated sequences, we utilized Gibson cloning (NEB) on the pLV-ICAM2-mKate construct. Each mutated construct contained mKate as the FP and the ICAM2 signal peptide, which is amino acids Met1 to Gly21. ICAM2 Δ1 was truncated from Ser22 to Pro111, ICAM2 Δ2 was truncated from Pro112 to Tyr216, ICAM2 Δ1-2 was truncated from Ser22 to Tyr216, and ICAM-2 ΔC was truncated from His251 to Pro275.

### Antibodies

Alexa Fluor 647-conjugated ICAM-1 mouse monoclonal antibody was purchased from AbD Serotec (MCA16115A647, IF 1 : 400). Anti ICAM-2 mouse monoclonal antibody was bought from Invitrogen (14-1029-82, IF 1:200). Alexa Fluor 647-conjugated chicken anti-mouse antibody was purchased from Invitrogen (A21463, IF 1 : 400). Alexa Fluor 488-conjugated chicken anti-mouse antibody was purchased from Invitrogen (A21200, IF 1 : 400).

### Cell culture

Cell culture was performed in a highly similar manner as described previously (11). HUVECs were obtained from Lonza (C2519A) and cultured on fibronectin-coated dishes in Endothelial Growth Medium 2 (EGM-2), supplemented with SingleQuots (Promocell, C-22011), and 100 U/mL penicillin and streptomycin (P/S), at 37°C in 5% CO_2_. Cells were cultured up to passage 7, and before the start of the experiment, they were not allowed to grow beyond 70% confluency.

CRISPR-control, ICAM1 KO, ICAM2 KO, and ICAM-1/2 KO blood outgrowth endothelial cells (BOECs) were generated in a previous study (11) and were grown on 0.1% gelatin-coated dishes during outgrowth and during experiments, and were grown in EGM-2 supplemented with SingleQuots, 100 U/mL P/S, and 18% fetal calf serum (Bodinco, Alkmaar, The Netherlands) at 37°C in 5% CO_2_. To induce inflammation, both HUVECs and BOECs were treated with 10 ng/mL recombinant TNFα (Peprotech (London, UK), 300-01A).

HEK-293T (ATCC) cells were cultures in Dulbecco’s Modified Eagle medium (DMEM, Gibco, 41965-039) containing 10% fetal calf serum with 100 U/mL P/S. In HEK-293T cells, lentiviral particles containing pLV plasmids were generated by transfecting packaging plasmids with TransIT (Myrus, Madison, WI, USA), following the manufacturer’s protocol. The lentivirus-containing supernatant was harvested, filtered (0.45 µm), and concentrated with Lenti-X concentrator (Clontech, 631232) on the second and third days after transfection. Depending on the efficacy of the virus, virus was added to HUVECs or BOECs at a ratio of 1 : 250 to 1 : 500. When required, a 2-day 1.5 μg/mL puromycin (InvivoGen (San Diego, CA, USA), ant-pr-1) selection was performed. ECs were used in assays at least 72 h after initial transduction, and mycoplasma contamination tests were performed on all cells used in the study every 3 months.

### Neutrophil isolation

Isolation of primary human neutrophils was performed as described previously (11). Polymorphonuclear neutrophils were obtained from healthy volunteer donors who provided informed consent and adhered to the regulations set forth by the Sanquin Medical Ethical Committee, which were in line with Dutch legislation and the Declaration of Helsinki. All blood samples were processed within 2 h of donation. To isolate the neutrophils, whole blood was mixed with 5% tri-sodium citrate (TNC) in PBS and layered on top of 12.5 mL Percoll (1.076 g/mL). After a 20-min centrifugation at 800 ***g*** with a slow start and no brake, the monocyte- and lymphocyte-containing ring fraction was discarded, and the remaining cells were treated with 45 mL of ice-cold erythrocyte lysis buffer for 15 min. The erythrocyte lysis process was repeated once, with a centrifugation step at 500 ***g*** for 5 min at 4°C in between. The resulting neutrophils were centrifuged at 500 ***g*** for 5 min at 4°C, washed once with 30 mL of ice-cold PBS, and then resuspended in RT HEPES medium (20 mM HEPES, 132 mM NaCl, 6 mM KCl, 1 mM CaCl_2_, 1 mM MgSO_4_, 1.2 mM K_2_HPO_4_, 5 mM glucose, and 0.4% (w/v) human serum albumin, pH 7.4). Neutrophil counts were determined using a cell counter (Casey), and the cells were maintained at a concentration of 2 million/mL at RT. Neutrophils were used within 4 h of isolation.

### Neutrophil transmigration under physiological flow

Neutrophil flow experiments were performed as described previously (11, 12). 20,000 BOECs per lane were seeded on collagen-coated Ibidi l-slides VI0.4 and grown for 48 h. TNFα treatment was administered 4 h prior to the experiment, when the ECs had formed a confluent monolayer. To label neutrophils, 6 million cells were incubated in Vybrant^TM^ DiD Cell-labeling solution (1 : 6000) at a concentration of 2 million cells/mL for 20 min at 37°C. The labeled neutrophils were washed by centrifugation, resuspended in HEPES medium, and allowed to recover at room temperature for 20 min. Next, 1 million neutrophils were incubated at 37°C for 20 min before use. The Ibidi flow chamber containing the ECs was connected to a perfusion system and subjected to a shear flow of 0.5 mL/min (0.8 dyne/cm^2^) for 2 min prior to injecting 700,000 neutrophils into the tubing system. To minimize the effect of neutrophil freshness on results, the order of samples was changed during the experiment.

Flow assays were imaged using an Axiovert 200 M widefield microscope, with a 10× NA 0.30 DIC Air objective (Zeiss) and a combination of a fluorescent excitation light source and a halogen lamp for transmitted light. Signal was detected with an AxioCam ICc 3 (Zeiss) camera. Images were taken every 5 s for 15 min in 2–4 positions in the middle of the ibidi flow chamber lane to analyze neutrophil crawling dynamics and diapedesis locations.

### Quantification of neutrophil transmigration dynamics

Imaris, version 9.7.2, was used to conduct all analyses. To evaluate the total adhesion and diapedesis efficacy, a spot analysis was on the end points in each field of view of each time-lapse video. To count the cells that adhered on the monolayer and the cells that crawled on the subendothelial side of the monolayer, spot analysis was carried out on the DiD channel with an estimated dot size of 10 µm. The spots were manually thresholded based on the spot quality filter in Imaris to differentiate neutrophils from background signal. To distinguish neutrophils above and below the endothelium, a filter based on intensity in the DIC channel was added to the pipeline. This filter could be used to count adhering and transmigrated neutrophils separately, as neutrophils are white and round when on top of the endothelium and black and spread out when underneath the endothelium. Total adhesion was calculated as the total number of neutrophils above and underneath the endothelium at 15 min. Neutrophil diapedesis efficacy was calculated as (Number of adhering neutrophils/Number of total detected neutrophils) × 100%. The same spot analysis on neutrophils above the endothelial layer was conducted on time-lapse data to assess neutrophil crawling dynamics.

A tracking step was added to the pipeline to connect the spots of each frame and detect neutrophil crawling patterns. The auto-regressive motion was used for tracking analysis, with a maximum distance of 20 μm between spots and allowing a gap size of one frame. To remove rolling neutrophils from the dataset, tracks with less than four spots were filtered out. To ensure optimal results, no more than 200 neutrophil tracks were permitted per video, and all tracks were manually checked for accuracy. Additionally, to only study neutrophils with a pronounced crawling phase, all crawling events under 20 μm in length were filtered out. From this analysis, the first and final location of each crawling event was determined, and tracks could be tallied either as an upstream or downstream crawling track. Compass plots of overlaid tracks were generated in Imaris.

Diapedesis site randomness was calculated as described before (11), but instead of using the first points of each crawling track, here all last points of each crawling track were isolated and masked onto one frame. Only tracks ending in successful diapedesis were used in this analysis. These points annotate diapedesis sites. A spot analysis was performed on this frame, and the average distance of each diapedesis site to each three nearest neighbors was calculated. In FIJI (v.1.52p) (30), the same number of random dots was generated and the same parameter of average distance to three nearest neighbors was calculated. As a measurement of diapedesis site randomness, the median distance to three nearest neighbors of random spots was divided by the median distance to three nearest neighbors of the actual diapedesis spots. The higher this parameter, the more random the diapedesis spots are distributed.

### Artificial hotspot neutrophil flow assay

Artificial hotspot neutrophils flow assays were performed as previously (11). ICAM-1/2 KO BOECs were transduced with truncated ICAM2 constructs and mixed with untransduced cells to create a mosaic monolayer. Neutrophil flow experiments were performed as described above. The DIC channel was imaged as described above. mKate was imaged with a 559–585 excitation filter, a 590 beam splitter, and a 600–690 emission filter, using an exposure time of 1.200 ms. Spots of neutrophil adhesion were manually analyzed for their location: on untransduced or on transduced ECs. Counted adhesion events were normalized against the percentage of the area in the field of view that was occupied by transduced cells.

### Immunofluorescent stains

HUVECs or BOECs were cultured in Ibidi l-slides VI0.4, coated with respectively FN and collagen. Fixation was done using 4% Paraformaldehyde (PFA) in PBS++, without the need for a permeabilization step since all antibodies used bound to extracellular epitopes. Blocking was done using 2% BSA in PBS++. Primary antibodies were then added and incubated for 1 h at RT in PBS++, followed by the addition of secondary antibodies, if necessary, which were also incubated for 1 h at RT in PBS++. After each fixation, blocking, and staining step, the flow chamber was washed three times with PBS++. In cases where two primary antibodies were raised in the same species, a three-step staining method was used. This involved starting with an unconjugated primary antibody, then adding an accompanying secondary antibody, and finally adding a second directly conjugated antibody. Fixed samples were then imaged in high resolution using a Zeiss LSM 980 with Airyscan 2 module, using a Plan-Apochromat 40× NA 1.3 oil DIC objective and a voxel size of 0.053 × 0.053 × 0.220 nm to capture Z-stacks. To detect specific labels, Alexa Fluor 488 was excited using a 488 nm laser, mKate was imaged using a 561 nm laser, and Alexa Fluor 647 was excited with a 639 nm laser. Maximum projections were constructed in FIJI after acquiring and processing 3D Airyscan images using Zen Blue, version 3.3, with Multiplex SR-8Y settings and a GaAsP-PMT detector being used as a detector.

### Statistical analysis

The data were presented as means or medians, along with the standard deviation, as indicated on each graph. To compare between two groups, a two-tailed *t*-test was performed. When relevant, a paired *t*-test was performed. For comparisons among multiple groups, a one-way ANOVA was used. For experiments with two conditions, a Student’s *t*-test or Mann–Whitney test was conducted. For experiments with multiple conditions, a one-way ANOVA was carried out, indicating the specific conditions being compared. A two-tailed *P*-value less than 0.05 was considered significant. Representative images were shown for microscopy data. No blinding procedures were used during the experiments.

## Results

### ICAM1 and ICAM2 play different roles during TEM

**Figure 1:**
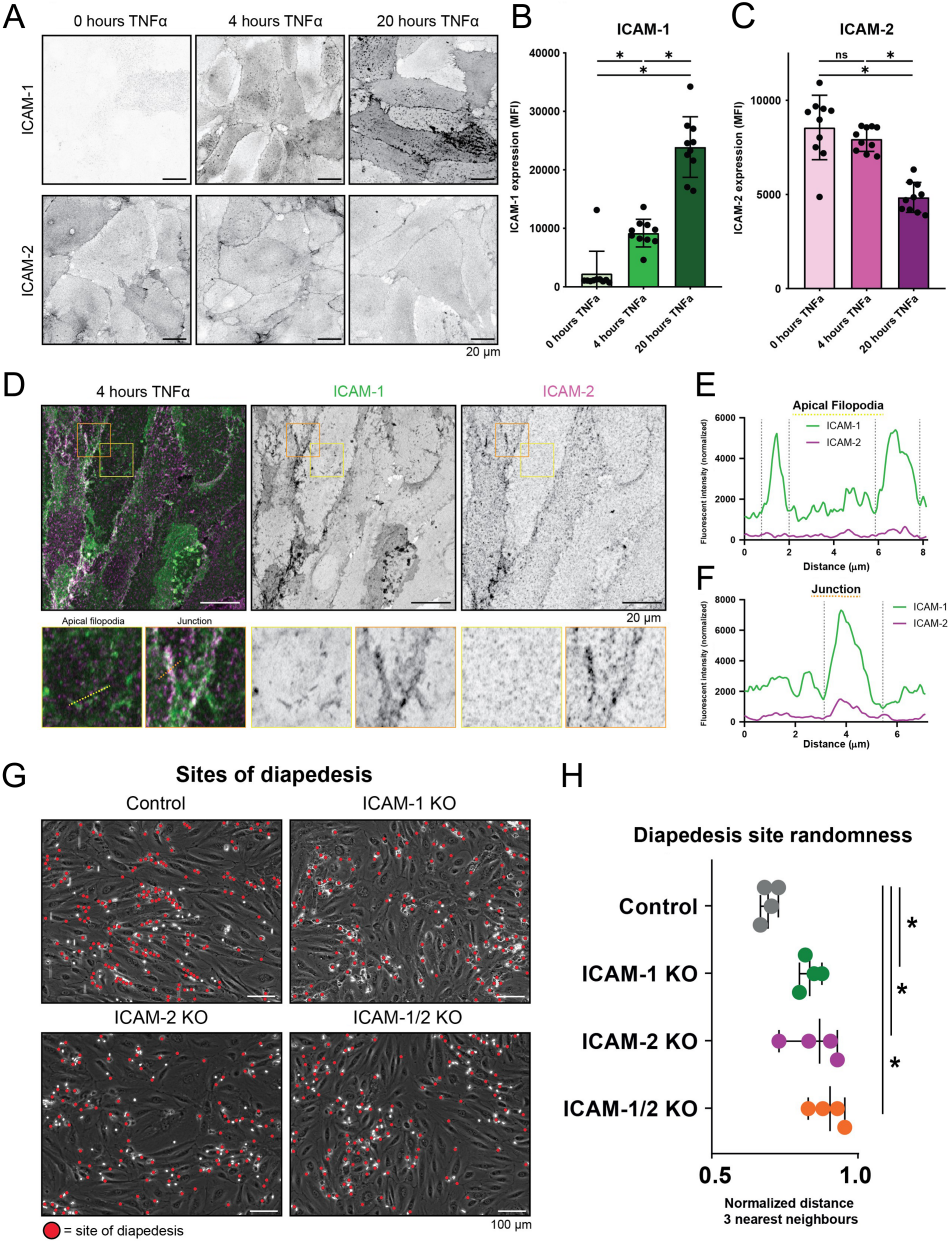
ICAM-1/2 KO increases diapedesis site randomness. (A) Inverted grayscale LUT of immunofluorescent stains for ICAM1 and ICAM2 on HUVECs treated with 0, 4, or 24 h of TNFα. Scale bar, 20 μm. (B) Bar graph displaying apical ICAM1 expression levels, measured by mean fluorescent intensity of the whole field of view. Each dot represents one field of view, and data are originated from 3 biological replicates. One-way ANOVA with multiple comparison was performed. 0 h vs 4 h: *P* = 0.0016. 0 h vs 24 h: *P* < 0.0001. 4 h vs 24 h: *P* < 0.0001. (C) Bar graph displaying apical ICAM2 expression levels, measured by mean fluorescent intensity of the whole field of view. Each dot represents one field of view (*n* = 10 for all), data are originated from 3 biological replicates. One-way ANOVA with multiple comparison was performed. 0 h vs 4 h: *P* = 0.4706. 0 h vs 24 h: *P* < 0.0001. 4 h vs 24 h: *P* < 0.0001. (D) Merged and inverted grayscale LUT of immunofluorescent costains for ICAM1 (green) and ICAM2 (magenta) on HUVECs treated with 4 h of TNFα. Scale bar, 20 μm. The yellow square displays a zoom of apical filopodia, and the orange square displays a zoom of a junction region. The yellow line is the region quantified in Fig. 1E and the orange line is the region quantified in Fig. 1F (E) Line graph displaying ICAM1 and ICAM2 intensity at the yellow dotted line in Fig. 1D, which was drawn over two apical filopodia (annotated between the gray border lines). (F) Line graph displaying ICAM1 and ICAM2 intensity at the orange dotted line in Fig. 1D, which was drawn over a junction (annotated between the gray border lines). (G) Phase-contrast stills from neutrophil flow time lapses of neutrophils over control, ICAM1 KO, ICAM2 KO, and ICAM-1/-2 KO BOECs. All neutrophil diapedesis sites that occurred during the time lapse are annotated with a red dot. BOECs, blood outgrowth endothelial cells. Scale bar, 100 μm. (H) Medians of average distance of diapedesis sites to three nearest neighbors, normalized against medians of the average distance to three nearest neighbors of the corresponding randomly generated spots. Data originate from 4 biological replicates. Paired one-way ANOVA was performed, comparing all conditions to the control. Control vs ICAM1 KO: *P* = 0.0253. Control vs ICAM2 KO: *P* = 0.0161. Control vs ICAM-1/2 KO: *P* = 0.0031.

ICAM1 is known to be a classic marker of endothelial inflammation (13), whereas ICAM2 is already present on resting endothelium (31). Additionally, it is known that ICAM1 and ICAM2 both serve as ligands for the leukocyte β2 integrins LFA-1 and MAC-1 (14, 15, 32), and both have been implicated in the rolling, firm adhesion, and diapedesis steps of the TEM cascade (2, 33). To study ICAM2 function in leukocyte TEM, we started by investigating ICAM1 and ICAM2 expression patterns in endothelial cells over the course of TNFα-induced inflammation. To measure specifically ICAM1 and ICAM2 that is expressed at the luminal/apical side of the endothelium, we performed immunofluorescent stains on nonpermeabilized HUVECs that were cultured on fibronectin-coated glass covers (Fig. 1A). As expected, ICAM1 expression increased significantly over the course of inflammation. At 20 h of TNFα, more ICAM1 was expressed compared to only 4 h of TNFα (Fig. 1B). In accordance to previous literature (34), ICAM2 expression was reduced on the apical side of the endothelial monolayer after 20 h of TNFα (Fig. 1C). To study ICAM1 and ICAM2 together, we continued treating HUVECs with 4 h of TNFα for all experiments described in this study. At 4 h of TNFα, we costained ICAM1 and ICAM2 and observed that whereas ICAM1 and ICAM2 where both present on the apical side of the endothelium, only ICAM1 localized at apical filopodia (Fig. 1D and E). As these endothelial structures have been implicated in the capture and firm adhesion of neutrophils (35), these data suggest that ICAM1 and ICAM2 play different roles in the TEM cascade. In line with this, we find both molecules enriched at junction regions (Fig. 1D and F), suggesting a potential role during the later stages of TEM. Previously, we showed that ICAM1, and not ICAM2, is involved in regulating adhesion hotspots (11). To study if ICAM1 and ICAM2 regulate randomness of diapedesis sites, we specifically studied the location where neutrophils exit the endothelium in endothelial cells that were depleted for ICAM1 or ICAM2, or both. Under control conditions, we found that neutrophils exit the endothelium at specific location, i.e. hotspots. When ICAM1, ICAM2, or both were depleted from the endothelium, neutrophils did not show any preference for specific spots to cross but rather showed a more random pattern to cross the endothelium (Fig. 1G and H). These data suggest that ICAM1 and ICAM2 both can determine where the neutrophil exits the endothelium.

### ICAM2 is not involved in neutrophil adhesion and diapedesis efficacy

**Figure 2:**
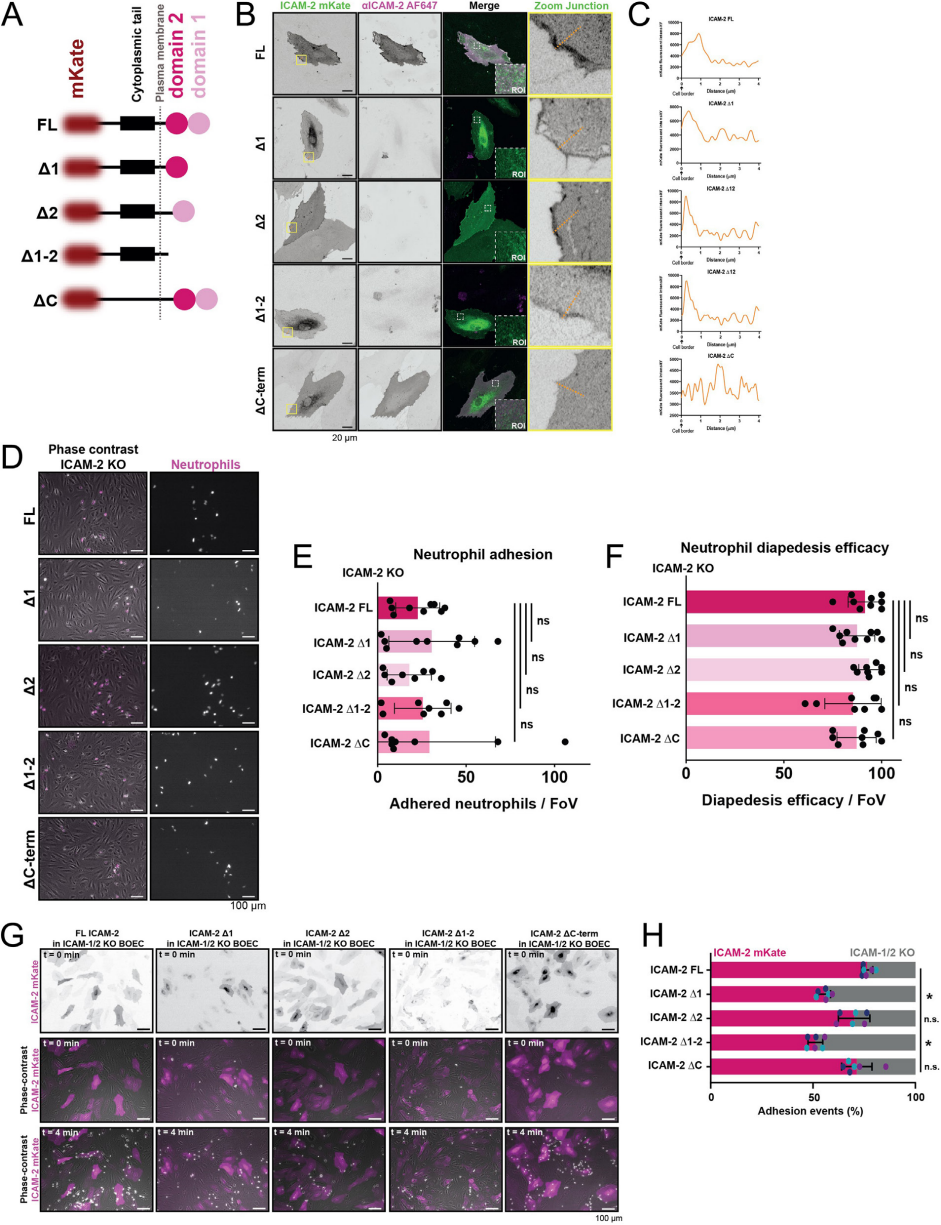
ICAM2 is not required for adhesion or diapedesis. (A) Schematic of all ICAM2 truncations used in this study. Note that the length of the black line does not correlate with the length of sequence between each domain. The plasma membrane is annotated with a gray dotted line. (B) Merge and inverted grayscale LUT of immunofluorescent stains for ICAM2 (magenta) on HUVECs treated with 4 h TNFα, which overexpress truncated ICAM2 constructs (green) Scale bar, 20 μm. White dotted squares show zooms of apical ICAM-2 signal, and yellow squares show zooms of junction areas. Orange lines are quantified in Fig. 1C (C) Line graphs displaying ICAM--mKate intensity at the orange dotted line in Fig. 1B. (D) Phase-contrast stills from neutrophil flow time lapses of neutrophils stained with DiD (magenta) over ICAM2 KO BOECs rescued with all ICAM2-mKate truncated constructs. BOECs, blood outgrowth endothelial cells. Scale bar, 100 μm. (E) Bar graphs displaying number of adhered neutrophils per field of view from neutrophil flow experiments in ICAM2 KO BOECs rescued with ICAM2-mKate truncated constructs. Each dot represents 1 field of view, and data are originating from three biological replicates. Mean and SD are shown. One-way ANOVA was performed, comparing all conditions with the FL rescue. FL vs Δ1: *P* = 0.8728. FL vs Δ2: *P* = 0.9781. FL vs Δ1-2: *P* = 0.9969. FL vs ΔC: *P* = 0.9344. (F) Bar graphs displaying neutrophil diapedesis efficacy per field of view from neutrophil flow experiments in ICAM2 KO BOECs rescued with ICAM2-mKate truncated constructs. Each dot represents 1 field of view, and data are originating from three biological replicates. Mean and SD are shown. One-way ANOVA was performed, comparing all conditions with the FL rescue. FL (*n* = 9) vs Δ1 (*n* = 9): *P* = 0.8070. FL vs Δ2 (*n* = 8): *P* = 0.9764. FL vs Δ1-2 (*n* = 8): *P* = 0.5319. FL vs ΔC (*n* = 8): *P* = 0.7882. (G) Time-lapse imaging of neutrophil TEM under flow with ICAM- 1/2 KO BOECs. A mosaic monolayer was created with untransduced ECs and ECs rescues with different ICAM2 truncations (magenta). Time is indicated in the top left. Scale bar, 100 μm. (H) Quantification of preferences for neutrophils to adhere to ICAM2-mKate truncations, expressed in ICAM-1/2 KO BOECs. Bars represent the percentage of neutrophils that adheres to transduced or untransduced cells. Numbers are corrected for the area occupied. Each dot represents a percentage of an individual time-lapse video, and each color represents one of three biological replicates. Means with s.d. are displayed.

To study ICAM2 distribution in more detail, we reexpressed ICAM2 mutants in ICAM2-deficient endothelial cells (Fig. 2A) and showed that ICAM2 tailless protein failed to localize at cell–cell junction regions (Fig. 2B and C). All other mutants, lacking parts of the extracellular domain localized to cell–cell junctions, as the full length ICAM2 did (Fig. 2B and C). To study if these mutants alter the adhesion or diapedesis of neutrophils in any way, we performed neutrophil TEM assays under physiological flow in ICAM2 knockout endothelial cells that were rescued with ICAM2 full-length and with all mutants as described above (Fig. 2D). No change in neutrophil adhesion numbers were observed in any of the mutants, indicating that ICAM2 is not involved in the adhesion step of TEM (Fig. 2E). Additionally, diapedesis efficacy was unaltered in any of the conditions (Fig. 2F). These data suggest that the intracellular domain of ICAM2 is responsible for its localization at junction regions and that ICAM2 is not involved in the direct adhesion or diapedesis of neutrophil under flow conditions in inflamed conditions, in line with previous research (11, 36).

However, ICAM2 does expose two Ig domains of which the first one serves as epitope for the neutrophil integrins LFA-1 and MAC-1. This may indicate that ICAM2 can compensate for ICAM1. To test if ICAM2 can solely mediate adhesion of neutrophils under flow conditions, we used endothelial cells that were depleted for ICAM1 and ICAM2. By rescuing ICAM2 full-length, we found that most neutrophils favored ICAM2-expressing endothelial cells (Fig. 2G and H). Moreover, when depleting the first Ig domain if ICAM2, we found reduced numbers of neutrophils adhering to these cells compared to ICAM2-deficient endothelial cells that were rescued with the full length ICAM2. Clearly, no additional role in mediating neutrophil adhesion was found for the second Ig domain or the intracellular tail (Fig. 2G and H). These data show that ICAM2 can compensate for ICAM1 in conditions where ICAM1 adhesive function may have been hampered.

### ICAM2 allows crawling against the direction of flow

**Figure 3:**
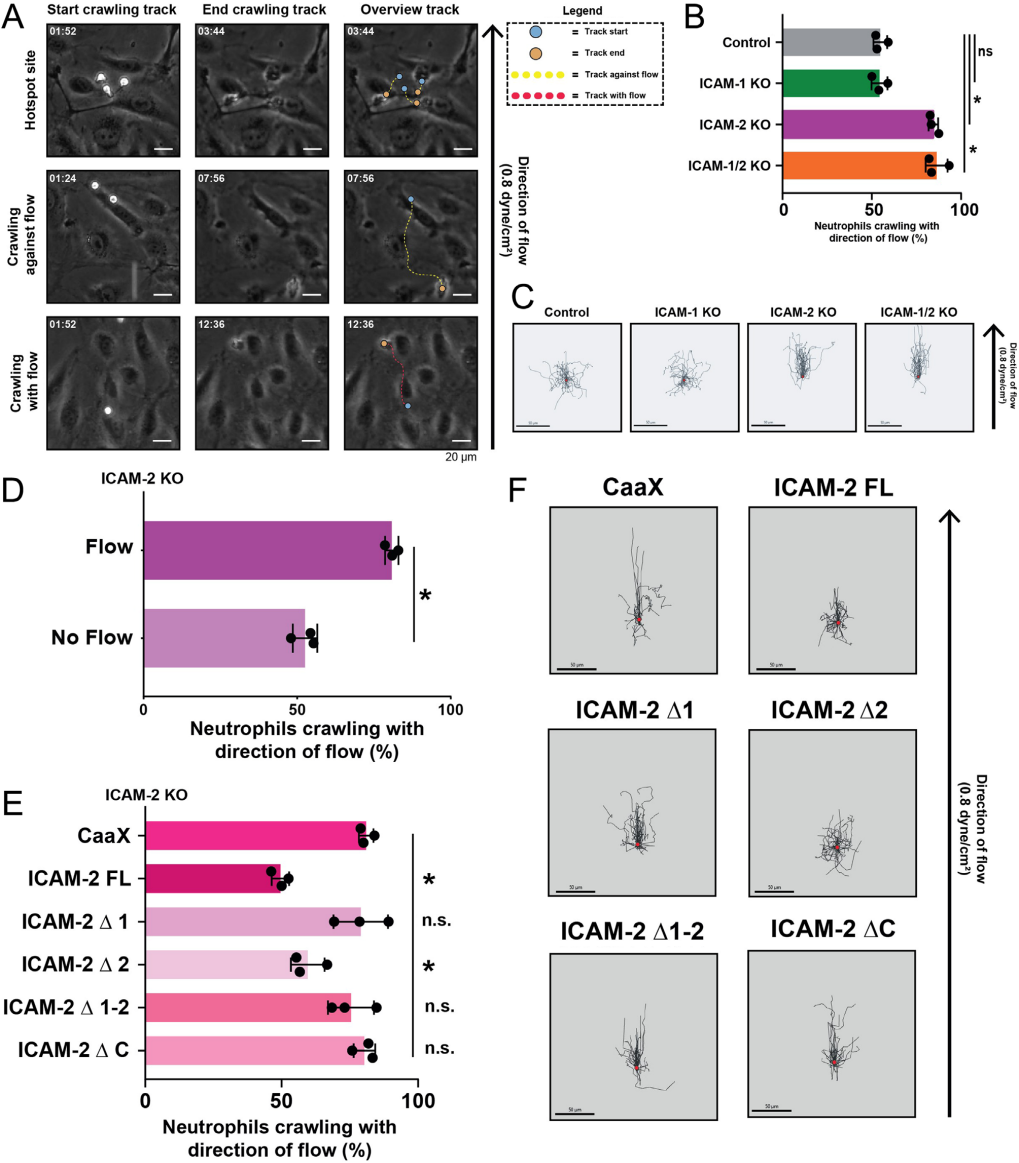
ICAM2 allows neutrophil crawling against flow. (A) Phase-contrast images from neutrophil flow time lapses of neutrophils. Examples of three different types of crawling behavior are displayed. The upper panels show an example of a hotspot site, the middle panels show an example of extended crawling against the direction of flow, and the lower panels show an example of extended crawling with the direction of flow. The left panels show the starting timepoints of each track, the middle panels show the end points of each track, and the right panels show an overview of the whole track. Blue dots show track starts, orange points show track ends, yellow dotted lines are tracks crawling against the direction of flow, and red dotted lines are tracks crawling with the direction of flow. The big black arrow shows the direction of flow. Scale bar, 20 μm. (B) Bar graph displaying directionality of neutrophil crawling tracks over control, ICAM1 KO, ICAM2 KO, and ICAM-1/2 KO BOECs. Dots correspond to mean percentages of neutrophils crawling with the flow from three biological replicates. The mean with s.d. is plotted. One-way paired ANOVA with multiple comparison correction, comparing all conditions with control, was performed on the means of each biological replicate. BOECs, blood outgrowth endothelial cells. Control (*n* = 324 tracks) vs ICAM1 KO (*n* = 569 tracks): *P* = 0.9990. Control vs ICAM2 KO (*n* = 529 tracks): *P* = 0.0009. Control vs ICAM-1/2 KO (*n* = 400 tracks): *P* = 0.0007. (C) 40 overlaid tracks of neutrophils crawling over control, ICAM1 KO, ICAM2 KO, and ICAM-1/2 KO BOECs. The big black arrow shows the direction of flow. Scale bar, 50 μm. (D) Bar graph displaying directionality of neutrophil crawling tracks over ICAM2 KO BOECs, where neutrophils were subjected to 0.8 dyne/cm^2^ shear flow (*n* = 111 tracks), or no flow (*n* = 118 tracks). Dots correspond to percentages of neutrophils crawling with the flow from three individual experiments. The mean with s.d. is plotted. A *t*-test was performed, *P* = 0.0004. (E) Bar graph displaying directionality of neutrophil crawling tracks over ICAM2 KO BOECs rescued with different ICAM2-mKate truncations or mScarletI-CaaX. Only tracks over 20 µm were used in the analysis. Dots correspond to percentages of neutrophils crawling with the flow from three individual experiments. The mean with s.d. is plotted. One-way paired ANOVA with multiple comparison correction, comparing all conditions with CaaX control. CaaX (*n* = 69 tracks) vs FL (*n* = 99 tracks): *P* = 0.0003. CaaX vs Δ1 (*n* = 159 tracks): *P*= 9940. CaaX vs Δ2 (*n* = 95 tracks): *P* = 0.0057. CaaX vs Δ1-2 (*n* = 110 tracks): *P* = 0.7298. CAAX vs ΔC (*n* = 76 tracks): *P* = 0.998. (F) 40 overlaid tracks of neutrophils crawling over ICAM2 KO BOECs rescued with ICAM2-mKate truncations or CAAX control. The big black arrow shows the direction of flow. Scale bar, 50 μm.

*In vivo* studies implicated ICAM2 in the crawling phase of murine neutrophils prior to diapedesis (19). Therefore, we hypothesized that the reason for increased randomness of diapedesis sites (Fig. 1G and H) may be explained by a deficit in neutrophil crawling. When observing migration tracks of crawling neutrophils on inflamed endothelium, we found a variety in crawling behavior. At hotspot regions, many neutrophils showed reduced crawling behavior, whereas other neutrophils that searched for hotspots crawled longer distances, independent of the direction of the flow (Fig. 3A). When analyzing neutrophils that had a very distinct crawling phase, meaning having a migration length of at least 20 μm, we found that in control conditions, 50% of neutrophils crawled against the direction of flow before finding an exit site, whereas the other 50% crawled with the direction of flow to find an exit site (Fig. 3B and 3C). No change in this migration and crawling behavior was found in ICAM1-deficient ECs (Fig. 3B and 3C). However, in both the ICAM2 KO and ICAM-1/2 KO endothelial cells, neutrophils failed to crawl against the flow and instead crawled almost exclusively with the direction of flow (Fig. 3B and 3C), indicating that ICAM2 is required for the crawling of neutrophils against the flow.

When comparing neutrophils crawling over ICAM2 KO ECs under flow to neutrophils crawling over the same ECs, but with the flow turned off when neutrophils adhered to the endothelium, we observed that neutrophils were again able to crawl in all directions (Fig. 3D).

To further investigate which domains of ICAM2 are required for neutrophil crawling against the direction of flow, we performed rescue experiments in ICAM2-deficient ECs by expressing ICAM2 full-length, all ICAM2 mutants as described above, and used membrane marker GFP-CaaX as a negative control. As expected, crawling directionality against the flow was rescued when expressing full-length ICAM2 (Fig. 3E and 3F). However, in all conditions where ICAM2 mutants were expressed lacking the first Ig-like domain (i.e. the epitope for LFA-1), neutrophils failed to crawl against the flow (Fig. 3E and 3F). ICAM2 Δ2 rescue did enable neutrophils to crawl against the direction of flow, indicating that the second Ig-like domain is not crucial in this process (Fig. 3E and F). Most strikingly, ICAM2 ΔC failed to rescue the crawling behavior, indicating that next to the first extracellular Ig domain, also the intracellular domain of ICAM-2 is involved in regulating the crawling behavior of neutrophils against the direction of flow (Fig. 3E and F). Together, these data show that both the first Ig-like domain and the intracellular domain of ICAM2 are required for neutrophil crawling against the direction of flow. This migratory behavior is required for neutrophil TEM to find the proper TEM hotspots.

## Discussion

The existence of TEM hotspots for neutrophil extravasation *in vivo* and *in vitro* have been described by different groups (10, 37). Recently, we found that TEM hotspots are marked by heterogeneous ICAM1 expression and function to maintain vascular integrity during leukocyte TEM (11). We additionally found that depletion of endothelial ICAM2 resulted in increased leakage during TEM, albeit to a lesser extent than when ICAM1 was depleted. In this study, we show that under control inflammation conditions, ICAM2 does not play a crucial role for the number of neutrophils that adhere to the endothelium. However, in conditions where ICAM1 function is perturbed, ICAM2 can compensate and control neutrophil adhesion. Additionally, ICAM2 has an important role in regulating the crawling phase of neutrophils over the endothelium under flow conditions, and thereby determines coordinated diapedesis at the same exit sites. Mechanistically, we show that the first extracellular Ig-like domain, as well as the intracellular domains of ICAM-2 are required for neutrophils to be able to crawl against the direction of flow.

Previous studies have described a role of ICAM2 in junction maturation (29). In line with other groups (19), we confirmed that ICAM2 indeed is enriched in junction regions, but we did not observe any obvious morphological defects on our monolayers that were depleted for ICAM2 in our phase-contrast images. We did not study junctional phenotype via immunofluorescent stains of adherens junctions (e.g. VE-cadherin) or tight junction (e.g. claudin 5) proteins, but based on the fact we previously did not observe basal leakage of 70 kDa dextran proteins through ICAM2-deficient ECs (11), we do not expect junction instability in matured ICAM2-deficient endothelial monolayers. Moreover, Amsellem and colleagues used thrombin on noninflamed murine endothelial cells in their *in vivo* permeability assays, which is a more severe permeability-inducer compared to neutrophil TEM, which by itself does not induce vascular leakage (38, 39, 40).

Intravital imaging of neutrophil TEM through ICAM2 KO ECs demonstrated, in line with our data, that murine neutrophil adhesion was not altered upon ICAM2 KO, but that efficiency of the crawling phase was decreased (19). However, this study did not report an effect on crawling against the directionality of flow or whether neutrophils transmigrated in a more random manner compared to control animals. Strikingly, a study in the BBB ECs showed that ICAM1 KO, and not ICAM2 KO, led to more crawling of murine lymphocytes with the direction of flow (6). Whether these opposite results compared to ours can be explained by differences of EC origin or leukocyte type would be an interesting approach for future experiments.

Our data show that both the first extracellular Ig-like domain, and the intracellular tail of ICAM2 are involved in allowing neutrophil to crawl against the direction of flow. The first Ig domain serves as an epitope for LFA-1 and MAC-1 integrins on the neutrophils (16, 17, 18). Previous work showed that LFA-1 is involved in firm adhesion whereas MAC-1 mediates intravascular crawling on the endothelium, and both steps contribute to efficient TEM (19, 21, 22). It is therefore not surprising that truncating this Ig domain of ICAM2 results in impaired crawling behavior. Based on our data, we cannot state whether it is LFA-1 or MAC-1 binding to ICAM2 that causes the crawling phenotype.

Additional functional experiments on ICAM2 domains revealed that the intracellular domain of ICAM2 is required for neutrophil crawling against the flow. This observation, combined with our finding that ICAM2 no longer localizes toward junctions when its intracellular tail is truncated, raises the questions whether it is the localization of ICAM2, its actin-modulating functions through the intracellular tail or both that are required for the crawling behavior of the neutrophils against the direction of flow. From ICAM1, it is recognized that clustering of ICAM1 results in the recruitment of actin-binding proteins that can create a strong actin network (41, 42, 43). It is generally accepted that this network serves as a substrate for the leukocyte to crawl on (44). It is tempting to think that a different type of actin network can also be mediated by ICAM2 clustering and that this may provide enough strength for the migrating neutrophil to withstand the forces of the flow and crawl against it, to find the proper exit site. An interesting approach for future research is therefore to generate an ICAM2 ‘adhesome’ dataset, similar to the ICAM1 adhesome dataset previously published by our group (45). Comparing these datasets could show which actin-adaptor proteins bind to ICAM2 and not to ICAM1. Performing knockout or knockdown experiments on such ICAM2 binding proteins and studying the ability of neutrophils to crawl against the direction of flow would be exciting experiments for future research. Additionally, swapping the intracellular domains of ICAM1 and ICAM2 could yield insights into why ICAM2 but not ICAM1 is required for neutrophil crawling against the direction of flow.

In conclusion, we demonstrate that ICAM2 is required for neutrophils to crawl against the direction of flow to start diapedesis in a coordinated manner.

## Declaration of interest

The authors declare that there is no conflicts of interest that could be perceived as prejudicing the impartiality of the research reported. J D van Buul is a senior editor of *Vascular Biology*. J D van Buul was not involved in the review or editorial process for this article, on which he is listed as an author.

## Funding

This work was supported by ZonMw – The Netherlands Organisation for Health Research and Development (Vici grant #91819632).

## Author contribution statement

MLBG: Conceptualization, Methodology, Validation, Formal Analysis, Investigation, Writing – Original Draft Preparation, Visualization. MET: Validation, Investigation, Visualization. MK: Investigation. JJGA: Conceptualization, Methodology, Validation, Formal Analysis, Investigation. JDB: Conceptualization, Methodology, Recourses, Writing – Review and Editing, Supervision, Project Administration, Funding Acquisition.
